# The sodium/glucose cotransporters as potential therapeutic targets for CF lung diseases revealed by human lung organoid swelling assay

**DOI:** 10.1016/j.omtm.2021.11.008

**Published:** 2021-11-24

**Authors:** Hiroyuki Hirai, Xiubin Liang, Yifei Sun, Yihan Zhang, Jifeng Zhang, Y. Eugene Chen, Hongmei Mou, Youyang Zhao, Jie Xu

**Affiliations:** 1 Center for Advanced Models for Translational Sciences and Therapeutics, University of Michigan Medical Center, University of Michigan Medical School, 2800 Plymouth Road, Ann Arbor, MI 48109, USA; 2 Program for Lung and Vascular Biology, Stanley Manne Children’s Research Institute, Ann & Robert H. Lurie Children’s Hospital of Chicago, Division of Critical Care, Department of Pediatrics, Northwestern University Feinberg School of Medicine, 303 E. Superior Street, Chicago, IL 60611, USA; 3 The Mucosal Immunology & Biology Research Center, Massachusetts General Hospital, 55 Fruit Street, Jackson 1402, Boston, MA 02114, USA

**Keywords:** cystic fibrosis, CFTR, lung organoids, sodium/glucose cotransporters, sotagliflozin, SGLT, inhibitor, human induced pluripotent stem cells (hiPSCs), drug discovery

## Abstract

Cystic fibrosis (CF) is a lethal autosomal-recessive inherited disease caused by mutations in the cystic fibrosis transmembrane conductance regulator (CFTR) gene. In the present work, we derived human proximal lung organoids (HLOs) from patient-derived pluripotent stem cells (PSCs) carrying disease-causing CFTR mutations. We evaluated the forskolin (Fsk)-stimulated swellings of these HLOs in the presence of CFTR modulators (VX-770 and/or VX-809) and demonstrated that HLOs respond to CFTR modulators in a mutation-dependent manner. Using this assay, we examined the effects of the sodium-dependent glucose cotransporter 1/2 (SGLT1/2) inhibitor drugs phlorizin and sotagliflozin on the basis of our findings that SGLT1 expression is upregulated in CF HLOs and airway epithelial cells compared with their wild-type counterparts. Unexpectedly, both drugs promoted dF/dF HLO swelling. These results reveal SGLTs, especially SGLT1, as potential therapeutic targets for treating CF lung diseases and demonstrate the use of PSC-derived HLOs as a preclinical tool in CF drug development.

## Graphical Abstract

**Figure:**
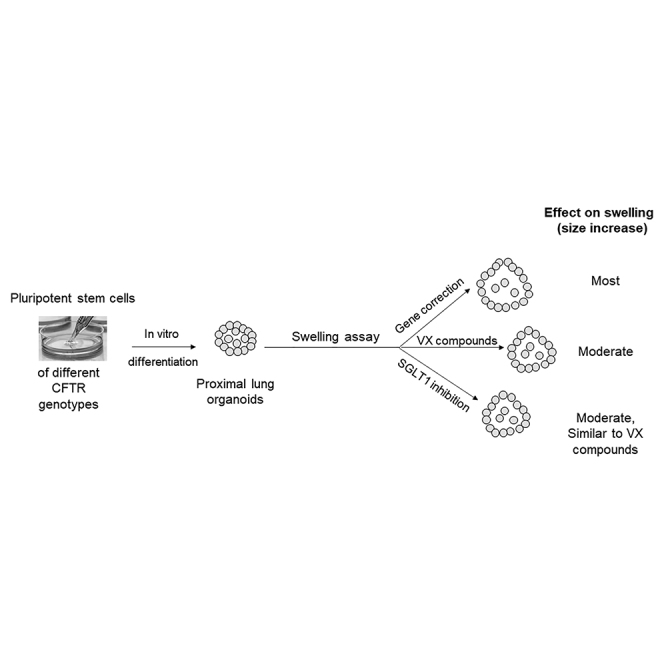

## Teaser

Proximal lung organoids were generated from induced pluripotent stem cells carrying different CFTR mutations. Using the swelling assay, we tested the effects of CFTR modulator drugs and SGLT1/2 dual-inhibitor drugs. The results suggest that SGLTs, especially SGLT1, are potential therapeutic targets for treating CF lung diseases.

## Introduction

Mutations in the cystic fibrosis transmembrane conductance regulator (CFTR) gene often lead to cystic fibrosis (CF), a lethal autosomal-recessive inherited disease.^1^ In October 2019, the U.S. Food and Drug Administration (FDA) approved Trikafta, which provides benefits to more than 90% of CF patients.^2^ Although the community celebrated this milestone achievement 30 years after the discovery the CFTR gene,^3^ the consensus remains that this marks a new start of our efforts in developing new and effective therapeutics for CF, as the disease is not cured yet.

Among animal and cellular models for the study of CF and CFTR, organoids have emerged as a model of choice. Organoids are three-dimensional (3D) *in vitro* cell cultures that can be derived using either primary cells or pluripotent stem cells (PSCs). For example, CF intestine organoids recapitulating essential features of the *in vivo* tissue architecture were first derived using patient primary intestine epithelial cells.^4^ More recently, in 2017, McCauley et al^5^ reported that Wnt signaling regulates lung differentiation of PSCs and that low-Wnt conditions allowed derivation of human proximal lung organoids (HLOs) from purified PSC-derived lung epithelial cells. These intestine and lung organoids, when subjected to a forskolin (Fsk)-stimulated swelling assay, respond in a mutation-dependent manner: normal CFTR wild-type (WT) organoids swell rapidly, whereas CF organoids show minimal expansion of size. Furthermore, the extent of swelling corresponds quantitatively with Fsk-induced anion currents.^4^ The swelling assay hence represents a simple and robust method to measure CFTR function in the organoids.

Sodium-dependent glucose cotransporters 1 and 2 (SGLT1/2) belong to the family of glucose transporters, encoded by SLC5A1 and SLC5A2,^6^ respectively. SGLT2 is almost exclusively expressed in the apical membrane of the renal proximal convoluted tubule cells, a site that is minimally affected in CF. SGLT1 is also expressed in the kidneys, but unlike SGLT2, SGLT1 is additionally expressed in many other tissues, including CF-relevant ones such as the lungs and the intestine. In a recent work, we observed elevated glucose absorption in the CF rabbit intestine, which can be blocked by the SGLT1/2 dual-inhibitor drug phlorizin (PHL), suggesting a role of SGLTs in CF.^7^ However, the effects of SGLT inhibitors on CF have not been tested.

In the present work, we derived HLOs using CF and non-CF PSCs. We demonstrated the application of these HLOs in preclinical research by testing the effects of the CFTR modulator drugs VX-770 and VX-809 and the SGLT1/2 dual-inhibitor drugs PHL and sotagliflozin (Sota) using the swelling assay.

## Results

### Derivation of isogenic HLOs using CFTR gene-edited PSCs

We recently reported efficient gene editing on major CFTR mutations and generated an induced pluripotent stem cell (iPSC) line carrying the homozygous CFTR delF508 (dF/dF) mutation.^8^ By gene-editing this dF/dF line, we subsequently generated a homozygously corrected wild-type/wild-type (cWT/cWT) line and a line carrying heterozygous compound mutations of delF508 and G551D (dF/G551D).

**Figure 1:**
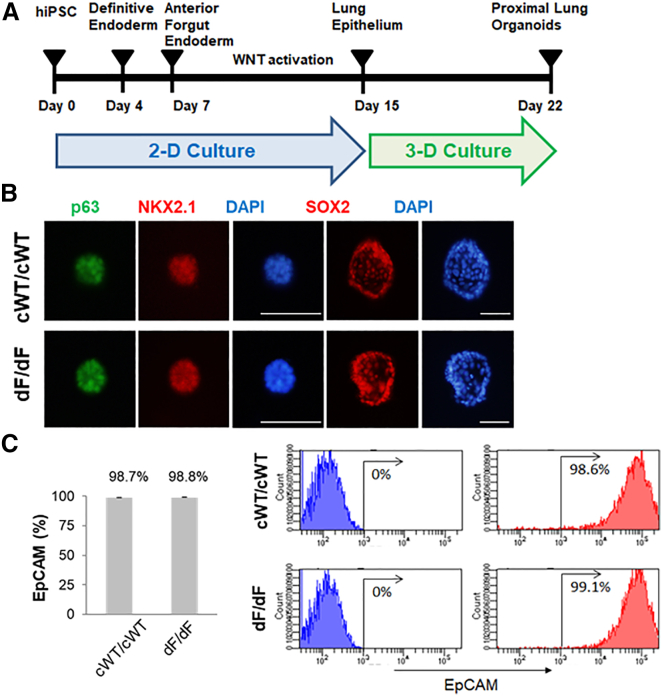
Generation of proximal lung organoids from cystic fibrosis patient iPSCs (A) Schematic representation of lung organoid differentiation protocol from human iPSCs (hiPSCs). (B) Representative immunofluorescence staining for p63 (green), NKX2.1 (red), SOX2 (red), and nuclear protein expression with DNA stain (DAPI; blue) in HLOs on day 20 (p63 and NKX2.1) and day 22 (SOX2). Scale bars represent 100 μm. (C) Flow cytometry analysis of EpCAM expression in organoid cells on day 23. Left: summary percentage of EpCAM-positive cells. Three independent HLO plates were used for the experiment. Data are presented as mean ± standard error of the mean (SEM). Right: a representative flow cytometry result. The x axis indicates the count of cells, and the y axis indicates EpCAM signal level.

Here we followed a 22 day regime (Figure 1A) described by McCauley et al.^5^^,^^9^ to establish HLOs *in vitro* using isogenic dF/dF and cWT/cWT iPSCs. Both dF/dF and cWT/cWT iPSCs were successfully differentiated into HLOs, following a similar course. By day 15 (D15), which marks the end of the two-dimensional (2D) culture period, most cells expressed lung epithelial progenitor markers, that is, CD47^+^high/positive and CD26^−^low/negative (CD47hi/CD26lo), evidenced by flow cytometry assays (Figure S1). After 1 week follow-up 3D culture, by D22–D23, sphere-shaped HLOs became evident (Figures 1B and S2). At this time, the HLOs, both dF/dF and cWT/cWT, were positive for p63, NKX2.1, Sox2, and MUC5AC, consistent with their fate into proximate lung lineage cells (Figure 1B). Flow cytometry assays showed that most cells now were positive for the epithelial cell marker EpCAM, again similarly in both cWT/cWT (98.7%) and dF/dF (98.8%) HLOs (Figure 1C). Interestingly a small percentage of cells expressed distal airway markers: 2.4% for alveolar epithelial type I (ATI) cell marker PDPN and 0.2% for ATII cell marker SFTPC, in both dF/dF and cWT/cWT HLOs (Figure S3).

These data show that gene-edited isogenic dF/dF and cWT/cWT iPSCs supported the derivation of isogenic HLOs.

### Gene correction restores CFTR channel function

We first worked to confirm that genetic correction of the CFTR mutation restores protein expression/maturation and leads to restoration of channel function. Indeed, cWT/cWT but not dF/dF HLOs expressed abundant functional CFTR proteins, evidenced by the mature CFTR band C in western blot assays (Figure S4A). When subjected to the swelling assay, the size of cWT/cWT HLOs increased by 2.12 times at 12 h and 3.54 times at 24 h, whereas dF/dF HLOs grew minimally (1.03- to 1.11-fold increase) during this period (Figures S4B and S4C).

### HLOs respond to CFTR modulators in a mutation-dependent manner

**Figure 2:**
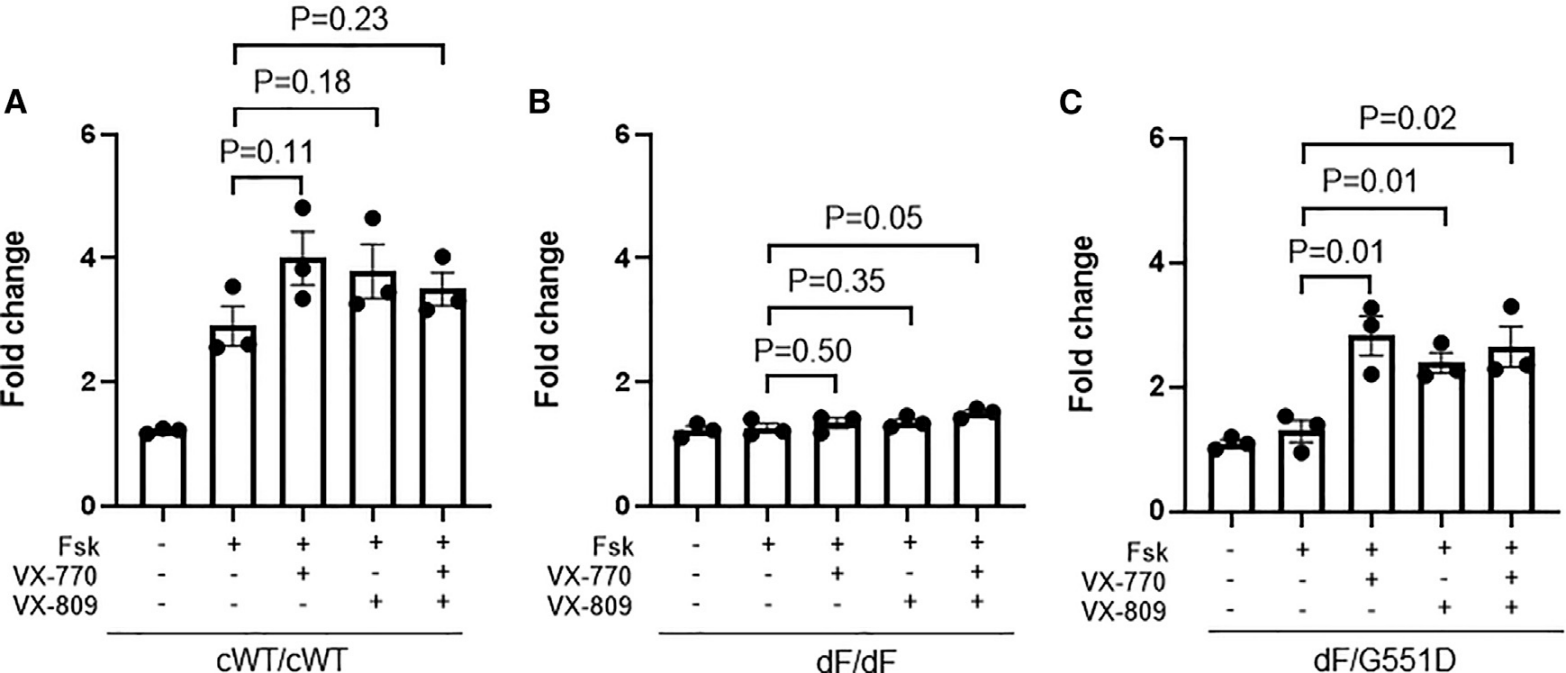
HLOs respond to VX compounds in a mutation-specific manner (A) Quantification of swelling of cWT/cWT HLOs treated with VX-770 and/or VX-809. (B) Quantification of swelling of dF/dF HLOs treated with VX-770 and/or VX-809. (C) Quantification of swelling of dF/G551D HLOs treated with VX-770 and/or VX-809. Data are presented as mean ± standard error of the mean (SEM). n = 3 biological replicates for each condition (from three wells of organoids derived from independent differentiation processes). Unpaired t test (two tailed) was used to compare data using GraphPad Prism 8 (GraphPad Software).

We proceeded to demonstrate the value of these HLOs in preclinical research. We first tested two FDA-approved compounds, VX-770 (a CFTR potentiator) and VX-809 (a CFTR corrector), on dF/dF HLOs. The combinational use of VX770 and VX809, but not VX-770 or VX-809 alone, led to a significant size increase of dF/dF HLOs (Figure 2B). We note that the effect size as indicated by the fold increase is modest: from 1.26 in the vehicle control group (i.e., without any VX drugs) to 1.50 in the VX-770 + VX-809 group. This is much lower than the extent of fold increase (2.90–4.00) in the cWT/cWT HLOs with or without VX treatment (Figure 2A), supporting the notion that genetic correction offers the most effective means for the restoration of CFTR function.

Because CF patients carrying the G551D allele benefit substantially from VX-770, we next tested if the swelling assay on CF HLOs carrying the G551D allele is reflective of patient response. The dF/G551D HLOs were established and subjected to the assay. As expected, VX-770 treatment to dF/G551D HLOs increased the size of swelling by more than 2-fold when it was applied alone or with VX809, significantly higher than that in the vehicle control group (Figure 2C).

These data demonstrate that the swelling assay on HLOs faithfully reflects the mutation-dependent response to CFTR modulators, supporting the use of this system in preclinical CF drug development.

### SGLT1 is upregulated in CF airway lineage cells and in CF HLOs

We next sought to use the CF HLO system to discover novel therapeutic targets for treating CF lung diseases. We selected SGLT1 as a target because some studies, including ours, have indicated increased SGLT1 activity in CF patients^10^ and in CF animals.^7^

First, we checked if SGLT1 is differentially expressed in CF airway lineage cells. We assessed its levels using western blot in CF bronchial epithelial (CFBE) cells, CF patient primary airway epithelial cells, and CF HLOs, in comparison with their non-CF counterparts.

**Figure 3:**
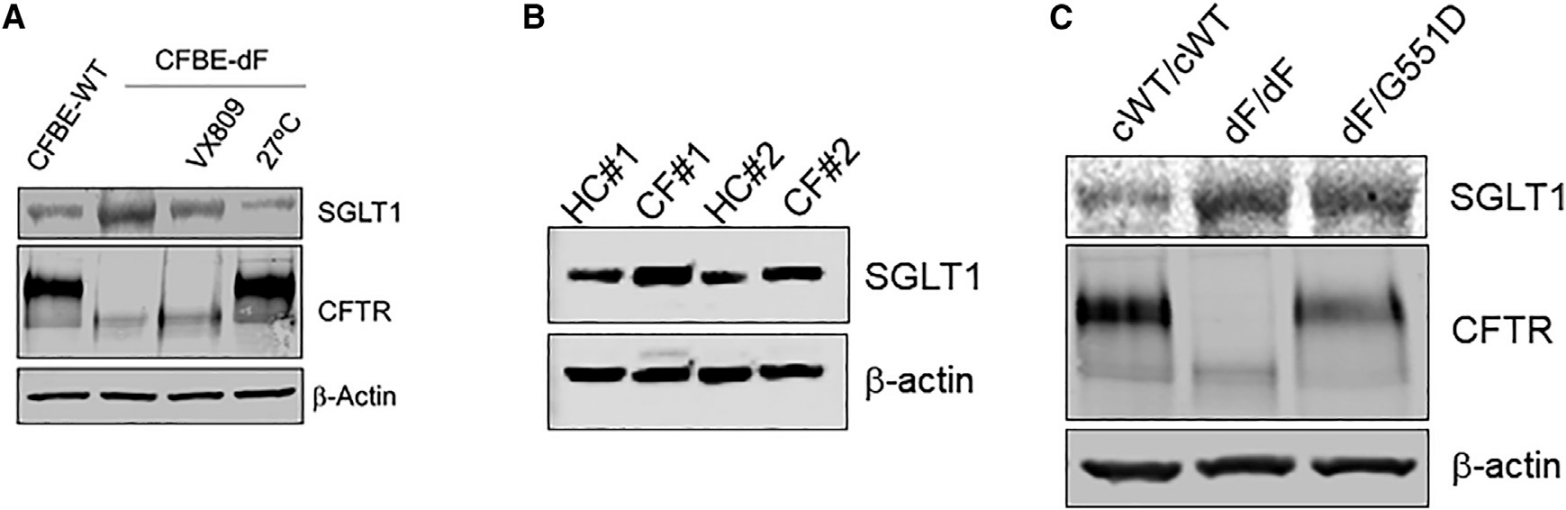
SGLT1 is upregulated in CF airway lineage cells and in CF HLOs (A) Western blot results in CFBE-WT cells (left lane) and in CFBE-dF cells under different conditions (three right lanes). VX-809, CFBE-dF cells treated with VX-809; 27°C, CFBE-dF cells cultured at 27°C. (B) Western blot results of matured airway epithelial cells derived from two dF/dF patients (CF#1 and CF#2) and from two healthy control subjects (HC#1 and HC#2). (C) Western blot results from cWT/cWT, dF/dF, and dF/G551D HLOs.

In the CFBE cells, the SGLT1 signals were reverse-correlated with those of CFTR: high in CFBE-dF cells but low in CFBE-WT cells (Figure 3A). Consistently, in CF patient primary airway epithelial cells (Figure 3B) as well as in CF HLOs (Figure 3C), the SGLT1 signals were higher than those from non-CF controls.

Intriguingly, pharmacological rescue by VX809 and low-temperature (27°C) rescue of CFTR in the CFBE-dF cells were both associated with reduced SGLT1 levels (Figure 3A).

Together, these data establish a robust reciprocal relationship between SGLT1 and CFTR. The consistent upregulation of SGLT1 in CF airway lineage cells promoted us to test the effects of SGLT1 inhibitor drugs in CF HLOs.

### Phlorizin and sotagliflozin promote dF/dF HLO swelling

All SGLT inhibitor drugs on the market are SGLT1/2 dual inhibitors^11^^,^^12^ (Table S1). In recent years, SGLT inhibitor drugs have gained extraordinary successes in treating diabetes and several diseases beyond diabetes.^11^ We are interested to know if this class of drugs has any potential to bring benefits to CF lung disease.

Here we selected PHL, sotagliflozin, and empagliflozin (Empa) to treat dF/dF HLOs in the swelling assay. PHL has 1:1 selectivity between SGLT2 and SGLT1. Sota has 20-fold higher selectivity for SGLT2 over that of SGLT1. These two represent the two most effective SGLT1 inhibitors. Empa, with 2,500-fold higher selectivity for SGLT2 over SGLT1, was used to represent the most effective SGLT2 inhibitor.

PHL (100 μM) and Empa (10 μM) concentrations used in the present work were based on a previous report.^13^ The Sota concentration (20 μM) was determined in a toxicity experiment, in which human airway basal cells were exposed to Sota at serial concentrations (0, 1, 2.5, 10, 25, and 100 μM). It was shown that Sota has no overt cellular toxicity if used at lower than 25 μM (Figure S5).

**Figure 4:**
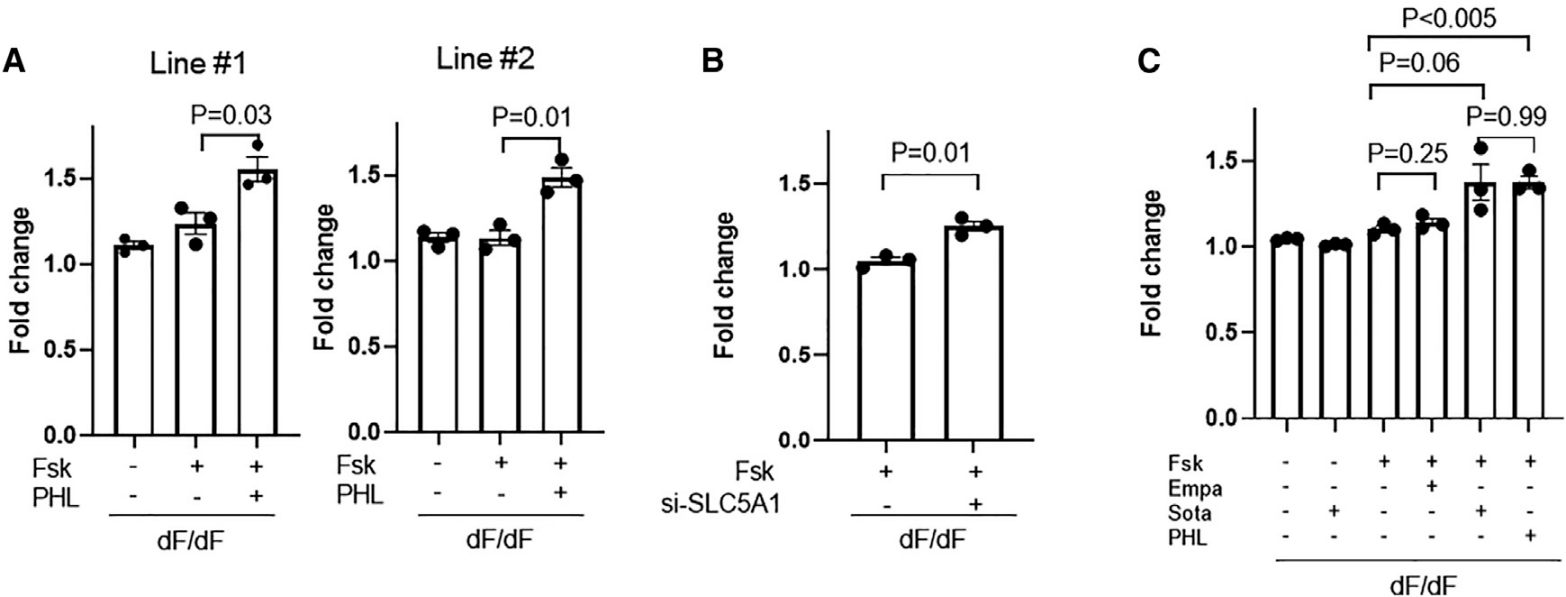
SGLT1/2 inhibitors promote CF HLO swelling (A) Effects of PHL on the swelling of dF/dF HLOs derived from two CF patient-derived iPSC lines. (B) Effects of siRNA targeting SLC5A1 on the swelling of dF/dF HLOs. (C) Effects of PHL, Sota, and Empa on the swelling of dF/dF HLOs. In organoid swell assays, n = 3 biological replicates for each condition (from three wells of organoids derived from independent differentiation processes). Unpaired t test (two tailed) was used to compare data using GraphPad Prism 8 (GraphPad Software).

PHL (100 μM) treatment led to swelling by 1.49- to 1.55-fold in two independent dF/dF HLO lines (Figure 4A), a similar extent to that achieved by VX-770/VX-809 (1.50) but significantly higher than that in the vehicle control group (1.14–1.24).

To confirm that the promoting effects by PHL are indeed through SGLT1 inhibition, we applied a small interfering RNA (siRNA) (si-SLC5A1) targeting the SGLT1 encoding gene SLC5A1 (Figure S6). As expected, a significant promoting effect was achieved by si-SLC5A1 but not by the non-targeting siRNA (Figure 4B).

Although PHL effectively promoted dF/dF HLO swelling, it is not a clinically approved drug. Hence, we next examined the effects of Sota, a drug that has gained European Union (EU) market approval for treating diabetes. In comparison, we included Empa, another SGLT inhibitor drug that is approved in the EU and the United States. Of note, Sota is >200-fold more effective on inhibiting SGLT1 (half maximal inhibitory concentration [IC_50_] = 36 nM) than Empa (IC_50_ = 8,300 nM).

In line with earlier data of PHL, Sota treatment (20 μM) to dF/dF HLOs promoted swelling by 1.38-fold (Figure 4C). In contrast, Empa treatment (10 μM) had minimal promoting effects (Figure 4C). Increasing the Empa concentration to 50 μM still had no effect (Figure S7A). Of note, Sota had modest effects on promoting WT HLO swelling (Figure S7B), which likely is due to the masking effects by the overall large fold of swelling in these organoids.

In the absence of Fsk, however, Sota has no effects on promoting dF/dF HLO swelling (Figure 4C), indicating that it works through a cyclic adenosine monophosphate (cAMP)-dependent mechanism.

To confirm the findings from the HLO system, we conducted the Premo Halide Sensor assay, a fluorescent protein-based method to measure channel activity that is commonly used in CF research,^14^ in the dF-CFBE and WT-CFBE cells (Figure S8). Consistent with the HLO swelling results, the decrease of fluorescence, an indicator of channel function, was most obvious in the WT-CFBE cells but least obvious in the vehicle control-treated dF-CFBE cells. Treatment of PHL and Sota to the dF-CFBE cells both led to approximately 20% fluorescence reduction, an effect size that is comparable with that on CF HLO swelling. These results confirmed the potential beneficial effects of PHL and Sota on CF airway cells.

Together, these data show that PHL and Sota treatment promoted CF HLO swelling through the inhibition of SGLT1.

### Mechanistic insights into SGLT inhibition in CF HLOs

**Figure 5:**
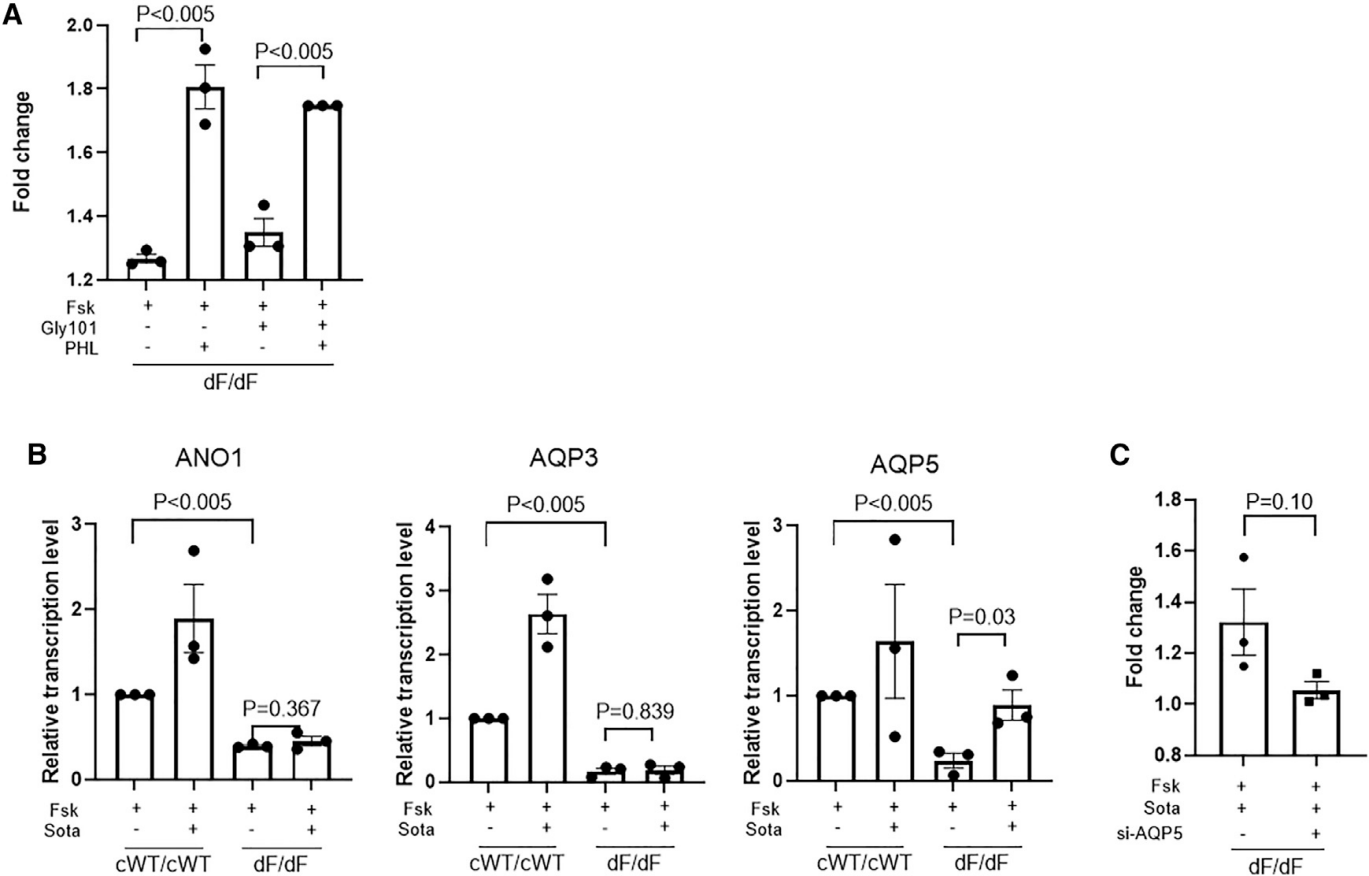
Mechanistic insights into the effects of PHL and Sota on dF/dF HLO swelling (A) Effects of GlyH-101 and PHL on the swelling of dF/dF HLOs. (B) Relative transcription levels of ANO1, AQP3, and AQP5 in cWT/cWT and dF/dF HLOs with or without Sota treatment. (C) Effects of siRNA targeting AQP5 on the swelling of dF/dF HLOs treated with Sota. n = 3 biological replicates for each condition (from three wells of organoids derived from independent differentiation processes). Unpaired t test (two tailed) was used to compare data using GraphPad Prism 8 (GraphPad Software).

We then worked to gain mechanistic insights into how Sota/PHL promotes CF HLO swelling. We first ask whether such effects are CFTR dependent. The co-application of GlyH-101, a CFTR inhibitor, did not affect the fold increase achieved by PHL (Figure 5A). This indicates that the swelling response of dF/dF HLOs to PHL is CFTR independent.

We next looked at several other channels that may potentially contribute to HLO swellings under SGLT inhibition. ANO1 (also known as TMEM16A) is a calcium-activated chloride channel (CaCC). Augment of ANO1 has led to promising clinical trials for treating CF patients who are not responsive to current modulator drugs.^15^ We also included a classic aquaporin (AQP5) and an aquaglyceroporin (AQP3), both of which are expressed mainly in airway cells. This is based on an earlier report in rats suggesting that SGLT1 inhibition activates AQP4 in epithelial cells.^16^

Quantitative RT-PCR (qRT-PCR) results revealed that in comparison with the cWT/cWT HLOs, the transcriptions of ANO1, AQP3, and AQP5 were all downregulated in dF/dF HLOs (Figure 5B). Although Sota treatment to dF/dF HLOs did not alter the transcription levels of ANO1 and AQP3, it had a significant elevating effect on that of AQP5 (Figure 5B). This finding suggests that AQP5 may have contributed to the Sota-promoted swelling in dF/dF HLOs. Indeed, the use of a siRNA targeting AQP5 (si-AQP5), but not a non-target siRNA, diminished the promoting effects by Sota (Figure 5C).

## Discussion

The present work demonstrates that PSC-derived HLOs may serve as a tool in CF drug development. When combined with gene editing, isogenic HLOs carrying different CFTR mutations can be generated. This is particularly useful for modeling rare mutations given the relatively scarce number of patients carrying mutations other than dF508.

We show that PSC-derived HLOs respond to CFTR corrector and potentiator compounds in a mutation-dependent manner. For example, CF HLOs carrying the G551D mutation responded well to VX770. VX809 treatment appears to elicit more benefits to heterozygous dF508 cells than to homozygous dF508 cells. Importantly, these observations are consistent with clinical findings.

Using this platform, we revealed promoting effects of PHL and Sota on CF HLO swelling, presenting inhibiting SGLTs as a potential new strategy to attenuate CF lung diseases. This finding has several implications. First, the promoting effects from PHL or Sota are likely achieved through inhibiting SGLT1 but not SGLT2, because the latter is minimally expressed in the lungs. This is further supported by the similar promoting effects achieved by the SLC5A1-specific siRNA. Second, we speculate that SGLT1 inhibition may bring benefits beyond the lungs, because it is expressed in multiple other CF-affected organ systems, including the intestines, the gallbladder, and the epididymis. Third, the promoting effects by SGLT1/2 inhibition are comparable with that achieved by combinational treatment with VX-770 (ivacaftor) and VX-809 (lumacaftor), the two compounds in Vertex’s Orkamibi, which was approved for treating dF/dF patients in 2015.^17^ Because the promoting effect appears to be CFTR independent, SGLT1 inhibitor drugs such as Sota may serve as a candidate “bridge” drug to treat CF patients (e.g., those of class I mutations) who currently do not benefit from Trikafta or any other drugs. Furthermore, although it is unlikely that Sota or PHL will exceed Trikafta in benefiting dF/dF, it is reasonable to speculate that a combinational use of Sota and Trikafta might bring synergistic effects. Nevertheless, future research should be conducted to verify and distinguish the effects of inhibiting SGLT1 and SGLT2 to test Sota as well as other SGLT-inhibiting reagents in other cellular systems and in preclinical animal models of different CFTR mutations and in clinical trials.

Mechanistically, our results, although preliminary, point to the potential contribution of AQPs when SGLTs are inhibited in CF. AQPs facilitate water transport across epithelia and play an important role in normal physiology and disease in the human airways.^18^ The link between SGLT1 inhibition and AQP, albeit in a non-CF model, was proposed in an early study using rat bile duct epithelial cells (i.e., cholangiocytes), in which the use of PHL activated AQP4.^16^ In CF conditions, it has been reported that CF airway epithelial cells express lower level of AQP3 compared with non-CF ones,^19^ a change that is similarly observed in our dF/dF HLOs. AQP5 is another AQP member that is expressed in the apical membrane of airway epithelial cells. Of note, it is cAMP dependent. It has been suggested that when substantially reduced, AQP5-mediated water transport can be a rate-limiting step in airway fluid secretion and that upregulation of AQP5 expression might reduce fluid viscosity in CF.^20^ In the present work, we provide support for this hypothesis: AQP5 is downregulated in CF HLOs, Sota treatment effectively elevated AQP5 transcription levels coinciding with the improved swelling assay results, and knockdown of AQP5 cancels Sota’s promoting effects.

One interesting technical observation is that PHL and Sota promoted HLO swelling despite the fact that they were added to the basal (i.e., outer) side of the organoids. This may indicate that the HLO epithelial cell junctions are leaky, such that PHL and Sota could diffuse into the apical (i.e., inner/lumen) side of the organoids to exert their effects. This view is supported by the promoting effects achieved by amiloride, a well-studied epithelial sodium channel (ENaC) inhibitor drug, when it was added to the culture medium (i.e., the basal side) of the HLOs (for 24 h) by us (Figure S9) and by Gibran et al.^21^ Like SGLT1, ENaC is expressed at the apical membrane.

Another interesting observation is that PHL and Sota have differential effects on SGLT1 protein levels. In dF/dF HLOs treated with Sota, but not those treated with PHL or Empa, the SGLT1 signals were lowered to a level comparable with those in the cWT/cWT HLOs (Figure S10). This raised the exciting possibility that Sota may enter the cells and exert its effects at the transcription and translation levels. This is particularly relevant given that we treated the HLOs with inhibitors for prolonged time (24 h). An RNA sequencing (RNA-seq) study with a focus on ion channels will provide valuable insights on Sota’s mechanisms of action.

One limitation of the present work is that we were not able to conduct more in-depth functional studies to delineate the roles of different channels and transporters (e.g., CFTR, SGLT1, ENaC). In a 2D cell culture system, such as CFBE cells, these are often studied using gold-standard Ussing chamber experiments.^22^ Unfortunately, 3D HLO cultures are not adaptable to the Ussing chamber yet.

We also want to point out that there is a small percentage of cells in HLOs (proximal lung organoids) that express distal lung cell type ATI (2.4%) and ATII (0.2%) markers. Therefore, we cannot exclude the possibility that these distal lineage cells contribute to the observed swelling phenotypes, especially given the observation that SGLT1 is expressed at higher levels in human alveolar cells than in tracheal epithelial cells.^23^

The concentrations of the compounds that we used in the present work may be further fine-tuned in a follow-up work. The PHL and Empa concentrations were based on an early study that used human embryonic kidney (HEK) cells.^13^ The Sota concentration was selected on the basis of the toxicity experiment on the human airway basal cells (Figure S5). Ideally the optimal concentrations of these compounds should be determined by a dose-response swelling experiment using the HLOs.

Last but not least, our work demonstrates that the most effective rescue, however, is genetic correction, underscoring the potential significance of gene editing on genetic diseases. In this regard, HLOs would serve as an ideal tool to bridge the conventional 2D cell culture platform toward *in vivo* therapeutics, which is in a 3D environment.

In sum, we report the establishment of PSC-derived isogenic CF HLOs, demonstrate the use of these HLOs for evaluating the effects of both FDA-approved and investigational compounds, and reveal SGLTs, especially SGLT1, as potential drug targets for treating CF lung diseases. Gene-edited HLOs may find broad applications in translational medicine.

## Materials and methods

### Cells

The human dF/dF iPSC line ACS-1004 was obtained from American Type Culture Collection (ATCC; Manassas, VA). cWT/cWT and dF/G551D iPSC lines were generated from ACS-1004 in a previous study.^8^ The human dF/dF iPSC line CF4 was obtained from the Cystic Fibrosis Foundation. CFBE-dF and CFBE-WT cells were gifts from Dr. Fei Sun’s laboratory at Wayne State University. CFBE-dF cells carry the CFTR-dF508 mutations, whereas CFBE-WT cells express wild-type CFTR.

### Definitive endoderm cell differentiation from iPSCs

Human iPSCs were harvested and triturated into single-cell suspensions with using Gentle Cell Dissociation Reagent (Stem Cell Technologies, Vancouver, BC, Canada) and seeded onto Corning Matrigel hESC-qualified Matrix (Corning, Corning, NY, USA) coated plate in mTesR1 (Stem Cell Technologies) containing 10 μM Y-27632 (Stem Cell Technologies) for 24 h. Then iPSCs were differentiated into definitive endoderm with using the STEMdiff Definitive Endoderm Kit (Stem Cell Technologies) for 72 h.

### Anterior foregut endoderm cell differentiation from definitive endoderm cells

Anterior foregut endoderm was differentiated from definitive endoderm cells and treated for 72 h with anterior foregut endoderm differentiation medium containing Ham’s F-12 Nutrient Mix (Thermo Fisher Scientific, Waltham, MA, USA) and IMDM (Thermo Fisher Scientific) with B27 Supplement (Thermo Fisher Scientific), N2 Supplement (Thermo Fisher Scientific), 0.1% bovine serum albumin fraction V (Millipore Sigma, St. Louis, MO, USA), 1-thioglycerol (Millipore Sigma), 1× GlutaMAX Supplement (Thermo Fisher Scientific), 1% penicillin-streptomycin, 50 μg/mL L-ascorbic acid, 10 mM SB431542 (Cayman Chemical, Ann Arbor, MI, USA), and 2 mM dorsomorphin (Cayman Chemical).

### Lung epithelial progenitor differentiation from anterior foregut endoderm cells

Anterior foregut endoderm cells were treated for 8 days with lung epithelial progenitor differentiation medium containing Ham’s F-12 Nutrient Mix and IMDM with B27 Supplement, N2 Supplement, 0.1% bovine serum albumin fraction V, 1-thioglycerol, 1× GlutaMAX Supplement, 1% penicillin-streptomycin, 10 ng/mL human recombinant BMP4 (Stem Cell Technologies), 50 μg/mL L-ascorbic acid, 3 mM CHIR99021 (Cayman Chemical), and 100 nM retinoic acid (Sigma-Aldrich).

### Proximal lung organoid differentiation from lung epithelial progenitors

On days 14 and 15, lung epithelial progenitors were dissociated into single-cell suspensions with trypsin-EDTA (0.05%) (Thermo Fisher Scientific). Harvested cells were immunostained with CD47 and CD26 antibodies. CD47^+^high/positive and CD26^−^low/negative cells were sorted, followed by resuspending as single cells in 50 μL three-dimensional growth factor reduced Matrigel drops, treated with proximal lung organoid differentiation medium containing Ham’s F-12 Nutrient Mix and IMDM with B27 Supplement, N2 Supplement, 0.1% bovine serum albumin fraction V, 1-thioglycerol, 1× GlutaMAX Supplement, 1% penicillin-streptomycin, 100 ng/mL human recombinant FGF10, 250 ng/mL human recombinant bFGF (Stem Cell Technologies), 50 μg/mL L-ascorbic acid, 100 nM dexamethasone (Cayman Chemical), 0.1 mM 8-bromo-Cyclic AMP (Cayman Chemical), 10 mM 3-isobutyl-1-methylxanthine (Sigma-Aldrich), and Y-27632 (Cayman Chemical).

### Measurement of Fsk-stimulated swelling of organoids

CFTR function was quantified by measuring Fsk-stimulated swelling of organoids as described previously.^5^^,^^8^ Organoids grown in Matrigel culture were passaged to new droplets at least 1 day prior to the swelling assay. In the assay, organoids were incubated with or without 10 μM Fsk (Selleck Chemicals), and swelling was monitored using time-lapse microscopy. To evaluate the effects of different compounds on the swelling, VX-770 (10 μM), VX-809 (3 μM), amiloride (10 μM), phlorizin (100 μM), Sota (20 μM), or Empa (10 μM) was added to the culture medium according to the experimental design 24 h before Fsk treatment. All compounds were purchased from Selleck Chemicals. The areas of individual HLOs at different time points were measured using ImageJ.^24^

### Knockdown of SLC5A1 and AQP5

Human SLC5A1 esiRNA (EHU020551) and AQP5 esiRNA (EHU046331) were purchased from Millipore Sigma. The cDNA target sequence, according to the manufacturer is shown below:

si-SLC5A1: 5′-ATCCAAGAAGGCCCTAAGGAGACCATTGAAATAGAAACACAAGTTCCTGAGAAGAAAAAAGGAATCTTCAGGAGAGCCTATGACCTATTTTGTGGGCTAGAGCAGCACGGTGCACCCAAGATGACTGAGGAAGAGGAGAAAGCCATGAAGATGAAGATGACGGACACCTCTGAGAAGCCTTTGTGGAGGACAGTGTTGAACGTCAATGGCATCATCCTGGTGACCGTGGCTGTCTTTTGCCATGCATATTTTGCCTGAGTCCTACCTTTTGCTGTAGATTTACCATGGCTGGACTCTTACTCACCTTCCTTTAGTCTCGTCCTGTGGTGTTGAAGGGAAATCAGCCAGTTGTAAATTTTGCCCAGGTGGATAAATGTGTACATGTGTAATTATAGGCTAGCTGGAAGAAAACCATTAGTTTGCTGTTAATTTATGCATTTGAAGCCAGTGTGATACAGCCATCTGTACCTACTGGAGCTGCAGAAGGGAAGTCCACTCA-3′
si-AQP5: 5′-TGGCTGCCATCCTTTACTTCTACCTGCTCTTCCCCAACTCCCTGAGCCTGAGTGAGCGTGTGGCCATCATCAAAGGCACGTATGAGCCTGACGAGGACTGGGAGGAGCAGCGGGAAGAGCGGAAGAAGACCATGGAGCTGACCACCCGCTGACCAGTGTCAGGCAGGGGCCAGCCCCTCAGCCCCTGAGCCAAGGGGGAAAAGAAGAAAAAGTACCTAACACAAGCTTCCTTTTTGCACAACCGGTCCTCTTGGCTGAGGAGGAGGAGCTGGTCACCCTGGCTGCACAGTTAGAGAGGGGAGAAGGAACCCATGATGGGACTCCTGGGGTAGGGGCCAGGGGCTGGGGTCTGCTGGGGACAGGTCTCTCTGGGACAGACCTCAGAGATTGTGAATGCAGTGCCA-3′

The negative control EGFP esiRNA (EHUEGFP) was also purchased from Millipore Sigma. HLOs were transduced with esiRNAs by Lipofectamine RNAiMAX Transfection Reagent (Thermo Fisher Scientific) with Opti-MEM (Thermo Fisher Scientific) following the manufacturer’s instructions. The swelling assay, in the presence of Fsk stimutation, was conducted 2 days post-transduction.

### Examination of SGLT1 on healthy control and CF human epithelial cells

Human adult airway basal cells were isolated from fresh discarded lung tissues at Massachusetts General Hospital under an institutional review board (IRB)-approved protocol (#2017P001479 and #2013P002332). Healthy control and CF airway basal cells (harboring CFTR-F508del/F508del) at their easiest passage were used to avoid the cell expansion-associated effects. Matured airway epithelial cells were generated on air-liquid interface using the protocol reported previously.^25^^,^^26^ The cell lysates were prepared from ALI culture at 16 days for western blot analysis to quantify SGLT1 expression (1:400-1:500, ab14686, Abcam, Cambridge, UK).

### Immunofluorescence staining

Cells were fixed with 4% paraformaldehyde (PFA) in PBS for 10 min and permeabilized with 0.5% Triton X-100 (Sigma-Aldrich) in PBS for 5 min at room temperature. Cells were stained with the primary antibodies for 1 h and secondary antibodies for 45 min each at room temperature. DNA was counterstained with DAPI. Primary antibodies included the following: TP63 (1:100, CM163A; Biocare Medical), TTF1 (1:200, ab76013; Abcam), SOX2 (1:200, 09-0024; ReproCELL USA Inc., Beltsville, MD, USA), PE-conjugated human EpCAM (1:200, 12-9326-42; Thermo Fisher Scientific), MUC5AC (1:200, MA1-38223; Thermo Fisher Scientific), APC-conjugated human CD47 (1:20, 323123; Biolegend), PE-conjugated human CD26 (1:20, 302705; Biolegend, San Diego, CA, USA), Alexa Fluor 647-conjugated human Podoplanin (1:200, 337007; Biolegend), and Alexa Fluor 488-conjugated human SP-C (1:200, sc-518029AF488, Santa Cruz Biotechnology, Dallas, TX, USA). Secondary antibodies included Alexa Fluor 488 goat anti-mouse IgG (H+L) secondary antibody (1:200, A11029; Thermo Fisher Scientific), Alexa Fluor 568 donkey anti-rabbit IgG (H+L) secondary antibody (A10042; Thermo Fisher Scientific), and Alexa Fluor 594 goat anti-mouse IgG (H + L) secondary antibody (A11032; Thermo Fisher Scientific).

### Western blot analysis

Equal amounts of proteins from CFBE-WT, -F508del, or organoids were resolved by SDS-PAGE and transferred to polyvinylidene difluoride (PVDF) membranes (PerkinElmer, Boston, MA, USA), which then were blocked at room temperature for 1 h with 5% (w/v) skim milk powder in TBST (10 mM Tris, pH 8.0), 150 mM NaCl, and 0.05% Tween 20. The blots were incubated with primary antibodies: anti-CFTR (catalog #217; Cystic Fibrosis Foundation, Bethesda, MD, USA) at 1:5,000, anti-SGLT1 (catalog #5042; Cell Signaling Technology, Danvers, MA, USA) at 1:1,000, or anti-beta-actin (catalog #3700S; Cell Signaling Technology) at 1:5,000 for 2 h at room temperature. After washing with TBST for 10 min and three times each with TBST, the blots were then incubated with horseradish peroxidase-conjugated secondary antibody for 1–2 h at room temperature on a rotating shaker. Western blots were scanned and quantified using an Odyssey Infrared Imaging System (version 2.1; LI-COR Biosciences, Lincoln, NE, U.S.A.).

### Premo Halide Sensor assay

The Premo Halide Sensor assay kit (P10229; Thermo Fisher Scientific) was used following the manufacturer’s instructions. Briefly, 5.5 mL Premo Halide Sensor transduction solution was added into the medium of CFBE cells, including WT, dF and dF CFBE pretreated with Sota (20 μM) or PHL (100 μM), respectively. The cells were cultured while being gently rotated at room temperature for 4 h. Then the enhancer solution (1:1,000 dilution) was added into the culture medium for 2 h at 37°C after removing the transduction solution. Next, cells were trypsinized into a 96-well plate for running the assay after cells were cultured in the normal medium overnight. Cells were stimulated with Fsk (10 μM) for 15 min and placed in a Premo Halide stimulus buffer containing iodide (150 mM NaI, 2.5 mM KCl, 1.8 mM CaCl_2_, 1 mM MgCl_2_, 10 mM HEPES, pH 7.4). The fluorescence baseline was recorded prior to the addition of NaI-containing buffer as time 0. The fluorescence was measured with a plate reader using 510–520 nm excitation wavelength 1, 3, 5, and 10 min after stimulation. The relative fold change of fluorescence quenching over that at 0 min was calculated to indicate CFTR channel function.

### Statistical analysis

Data are presented as mean ± SEM. For organoid swelling assays, n = 3 biological replicates (three wells of organoids derived from independent differentiation processes) were used. Student’s t test (two tailed) was used to compare data (GraphPad Software, San Diego, CA). p values ≤ 0.05 were considered to indicate statistical significance.

## Supplemental information

Document S1. Figures S1–S10 and Table S1

Document S2. Article plus supplemental information
