# Supplementary material for: The sodium/glucose cotransporters as potential therapeutic targets for CF lung diseases revealed by human lung organoid swelling assay
Source: Mol Ther Methods Clin Dev. 2021 Nov 24;24:11–9. doi: 10.1016/j.omtm.2021.11.008 (PMC8666609; doi:10.1016/j.omtm.2021.11.008)
Supplement: Supplementary file 1 — Document S1. Figures S1–S10 and Table S1 [file mmc1.pdf]

OMTM, Volume *24*

Supplemental information

The sodium/glucose cotransporters as potential therapeutic targets for CF lung diseases revealed by human lung organoid swelling assay

Hiroyuki Hirai, Xiubin Liang, Yifei Sun, Yihan Zhang, Jifeng Zhang, Y. Eugene Chen, Hongmei Mou, Youyang Zhao, and Jie Xu

Supplementary Data

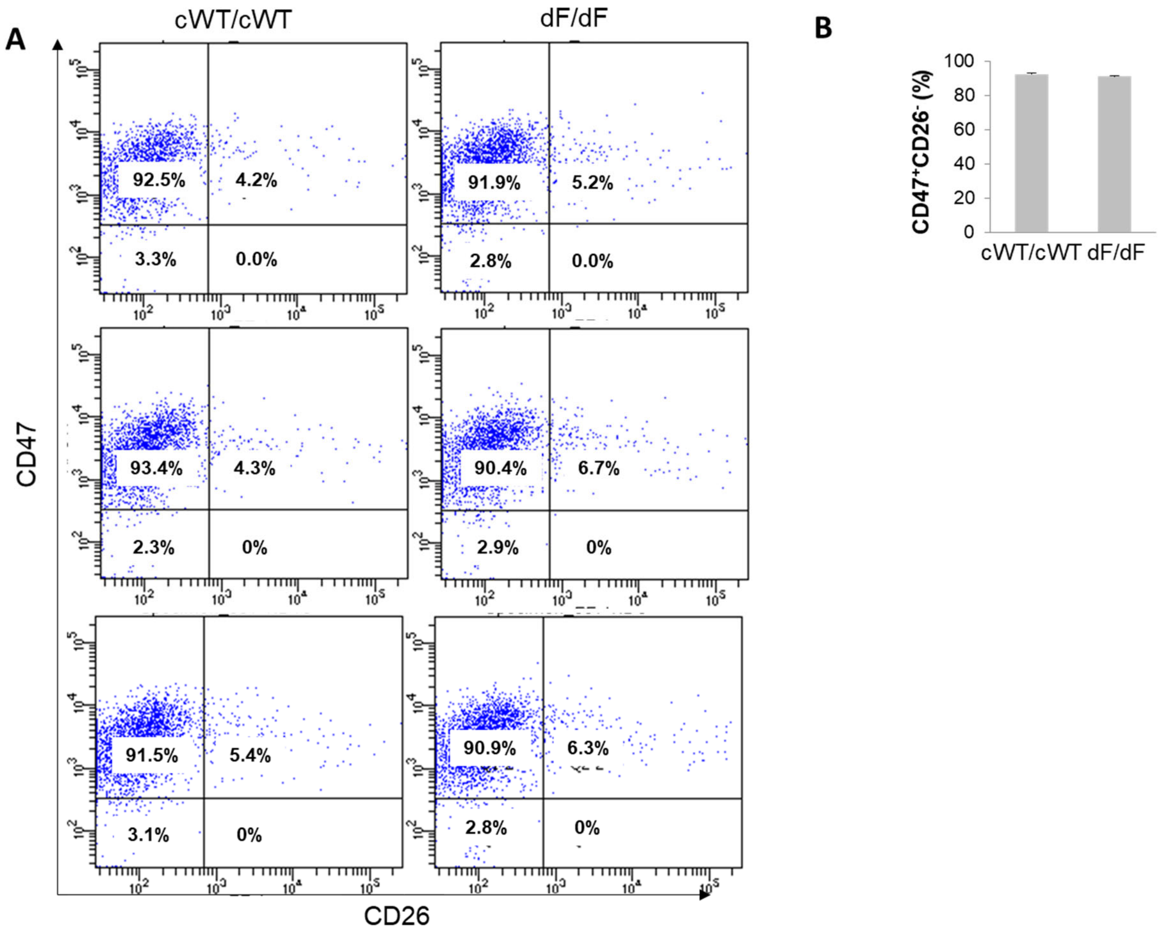

Figure S1. Generation of lung epithelial progenitor from CF patient derived iPSCs. (A) Representative flow cytometry result of CD47 and/or CD26 positive cells on Day 14-15 during HLO derivation process. CD47hi/CD26lo indicate lung epithelial progenitors. (B) Quantitative summary of CD47hi/CD26lo cell percentages from flow cytometry results.

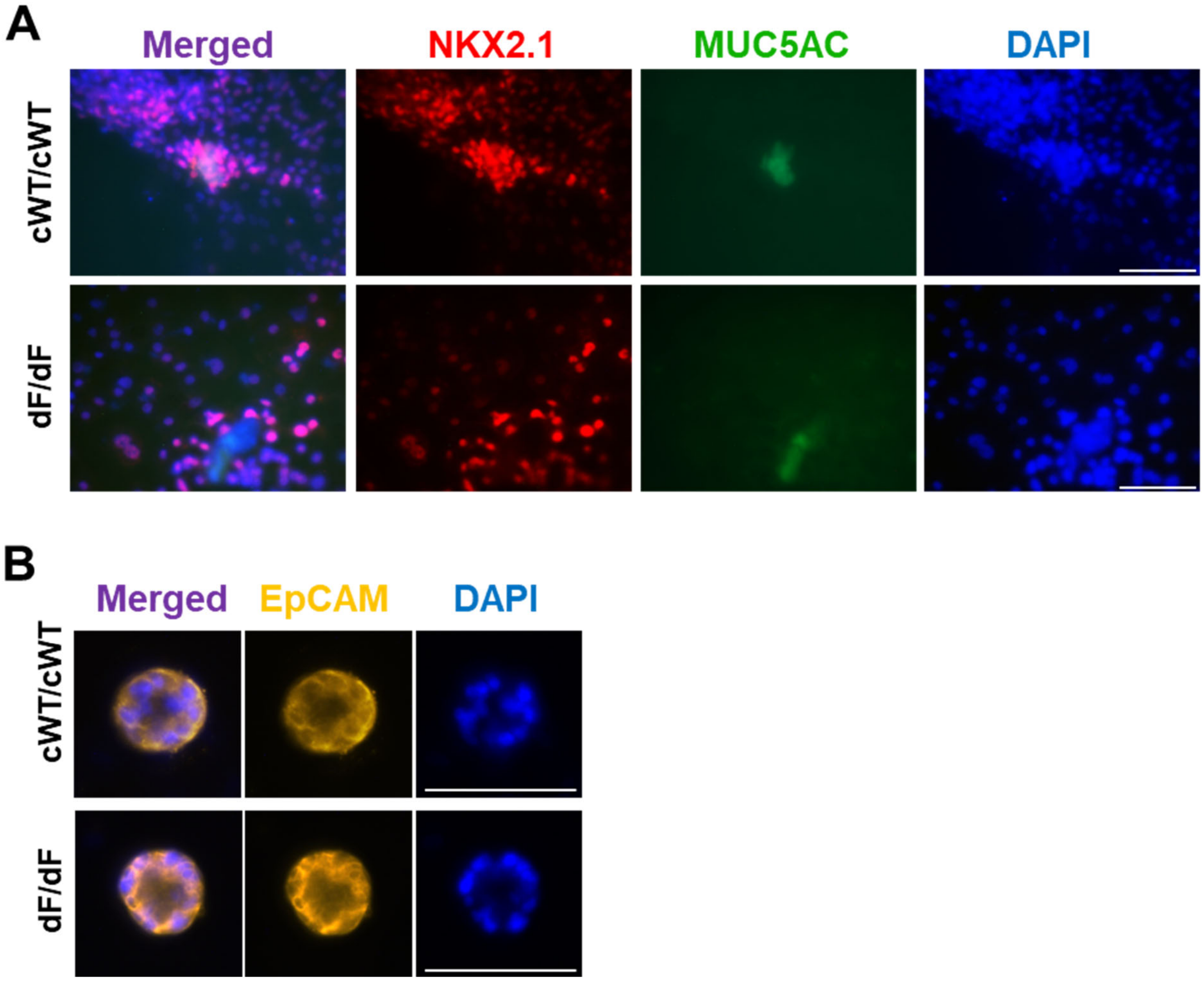

Figure S2. Generation of HLOs from CF patient derived iPSCs. (A) Representative immunofluorescence stains of NKX2-1 (red), MUC5AC (green), and nuclear protein expression with DNA stain (DAPI; blue) in D23 HLOs. Scale bars represent 100 µm. (B) Representative immunofluorescence stains of EpCAM (Orange) and nuclear protein expression with DNA stain (DAPI; blue) in D23 HLOs. Scale bars represent 100 µm.

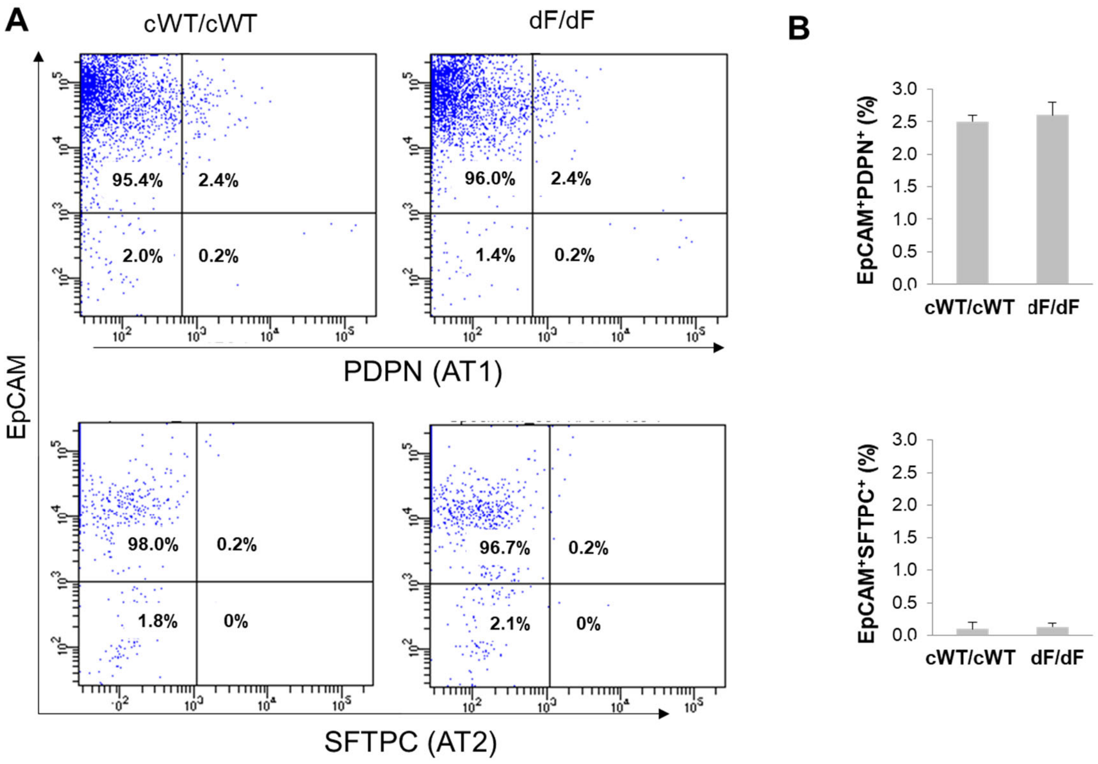

Figure S3. Low percentage of alveolar epithelial type I (AT1) and type II (AT2) cells in HLOs. (A) Representative flow cytometry result of $EpCAM^+$/$PDPN^+$ (AT1) and $EpCAM^+$/$SFTPC^+$ (AT2) in D23 HLOs. (B) Quantitative summary of $EpCAM^+$/$PDPN^+$ (AT1) and $EpCAM^+$/$SFTPC^+$ (AT2) percentages from flow cytometry results.

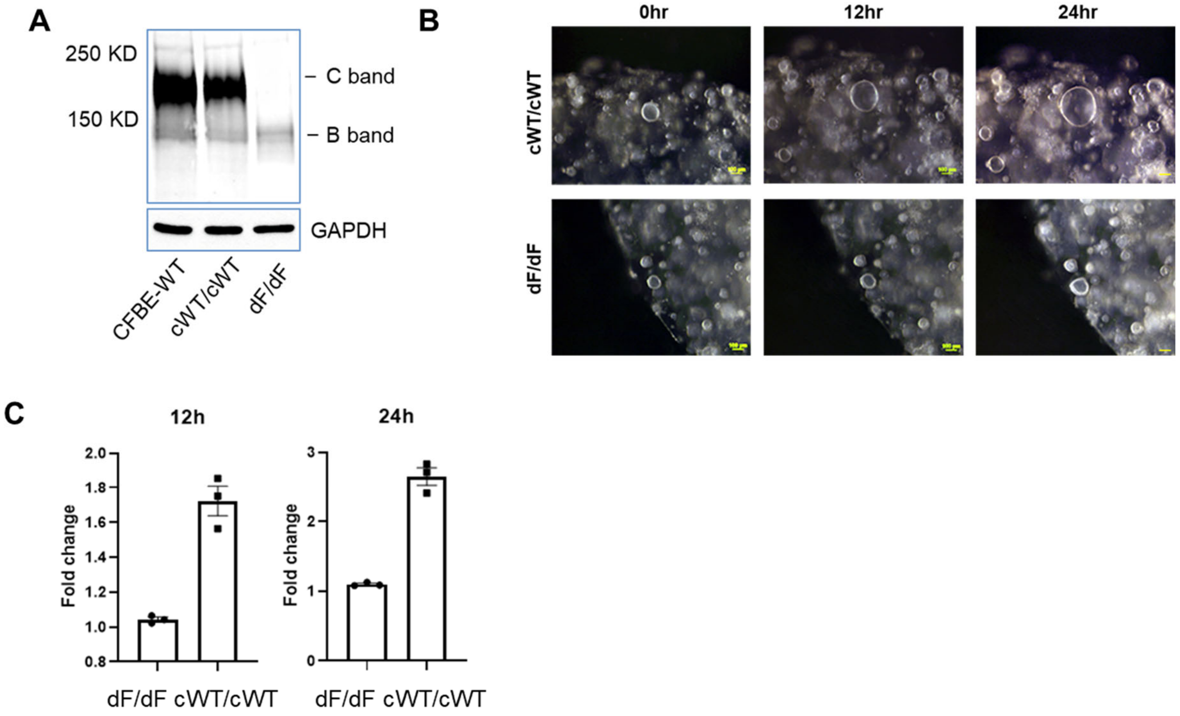

Figure S4. Gene correction restores Forskolin stimulated Proximal Lung Organoid swelling capacity (A) Western blot analysis of CFTR and GAPDH (loading control) in cWT/cWT and dF/dF organoids. CFBE-WT cells were used as positive Control. (B) Time-lapse phase contrast images of Fsk-induced swelling of cWT/cWT and dF/dF HLOs. Scale bars represent 100 µm. (C) Quantification of organoid swelling of cWT/cWT and dF/dF HLOs at 12h and 24h with Fsk stimulation.

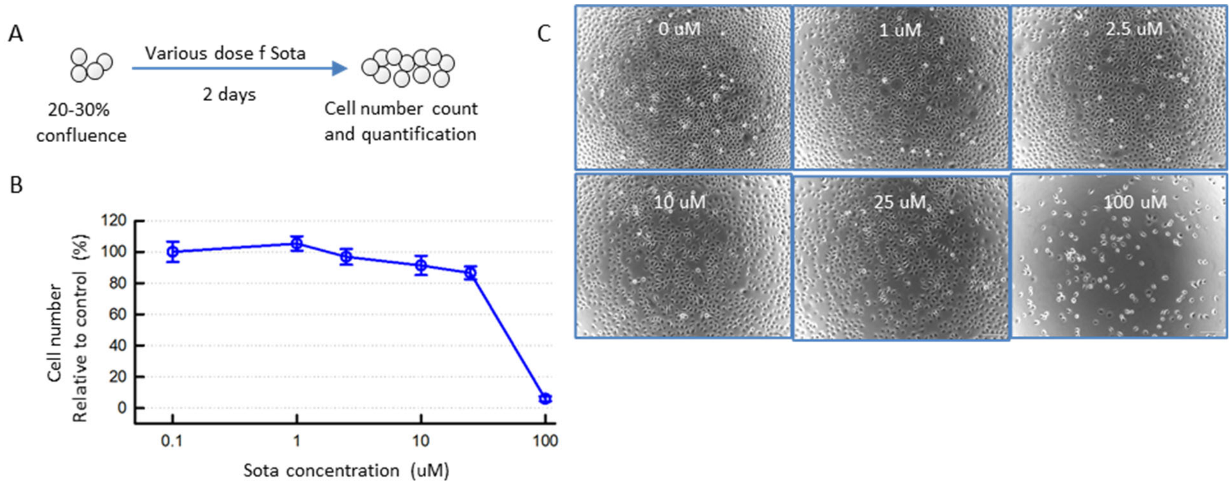

Figure S5. Determine toxic effects of Sota (LX4211) human airway basal cells. (A) Schematic illustration of the experimental strategy. In brief, human airway basal cells were cultured at initial seeding density of 20-30% in SAGM medium alone or medium containing various concentration of Sota for 2 days. The cells were then dissociated for total number counting. (B) The plot of total cell number relative to that without Sota treatment (n=3 of independent experiment). (C) The representative brightfield images of airway basal cells cultured in various doses of Sota.

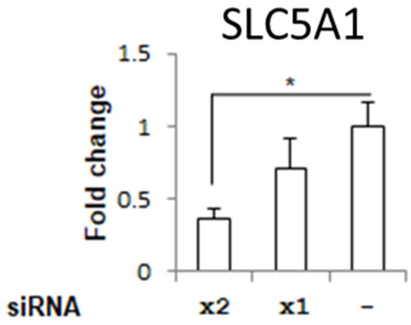

Figure S6. Knockdown of SLC5A1. Expression level of SLC5A1 in dF/dF HLOs achieved by two times transfections by SLC5A1 targeting siRNA (“x2”), one time transfection by SLC5A1 targeting siRNA (“x1”), or by a non-targeting siRNA (“-“).

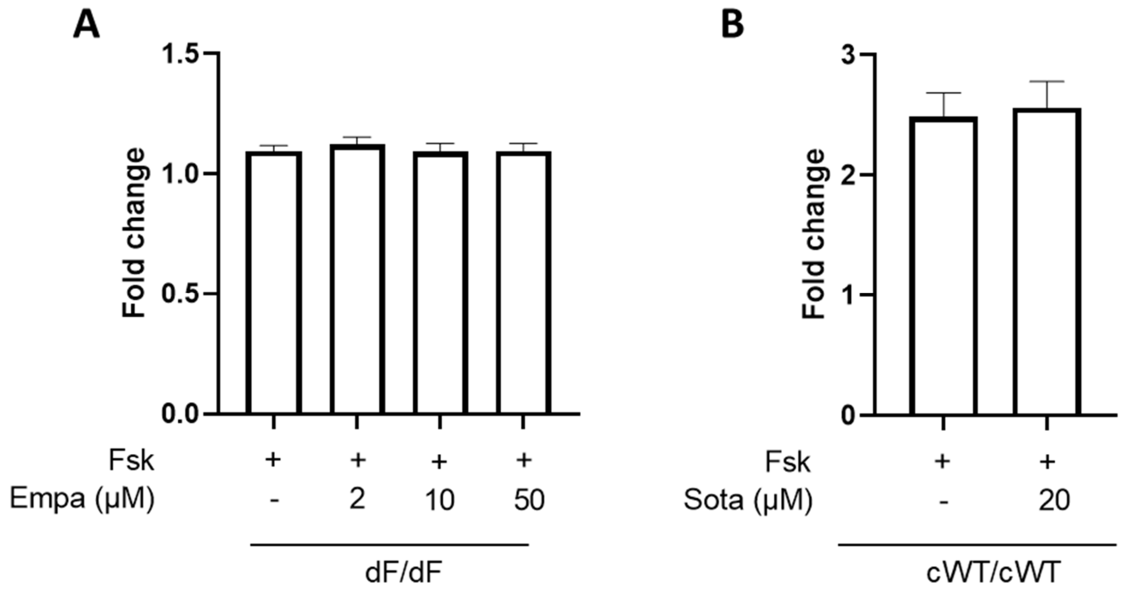

Figure S7. Quantification of organoid swelling. (A) dF/dF HLOs treated with different concentrations of Empa. (B) cWT/cWT HLOs treated with Sota.

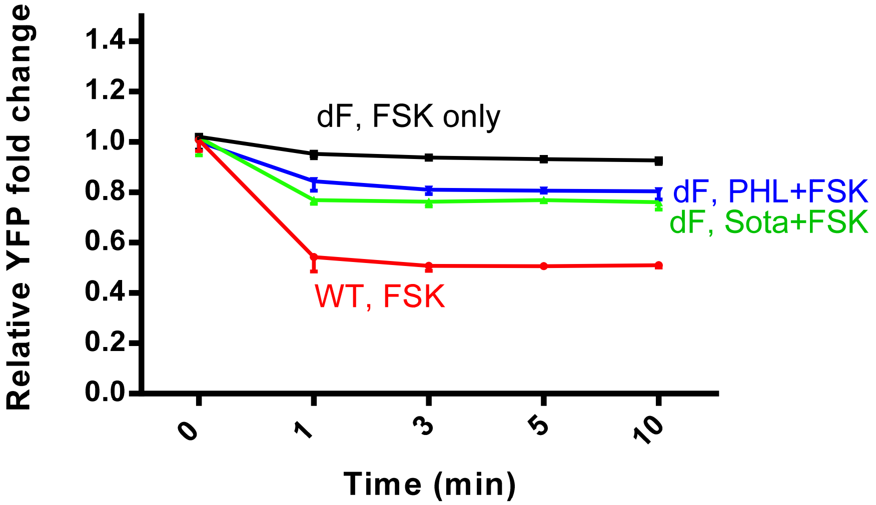

Figure S8. Relative fold change of YFP signals of CFBE-WT (red) and CFBE-dF cells in the Premo Halide Sensor Assay. CFBE-dF cells were treated with Fsk only (black) or Fsk with PHL (blue) or Sota (green). N=3 replicates using different plates of WT or CFBE-dF cells.

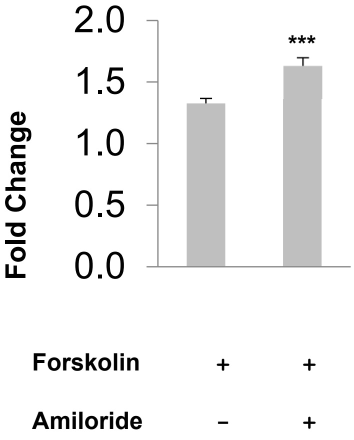

Figure S9. Effects of Amiloride on dF/dF HLO swelling. *** P<0.005

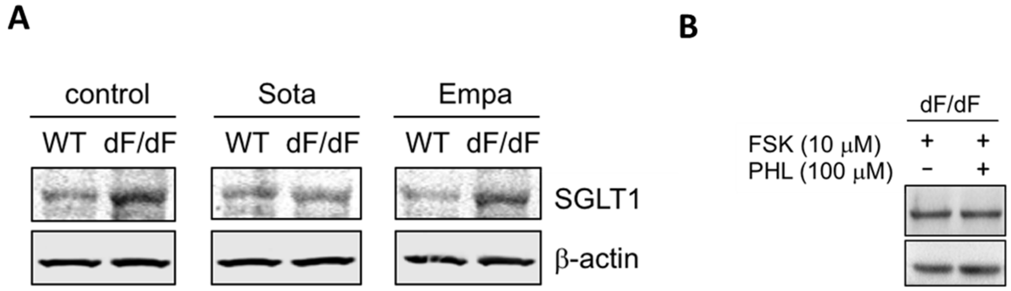

Figure S10. Effects of SGLT inhibitors on SGLT1 protein levels in HLOs. (A) Western blot results of SGLT1 levels in cWT/cWT (WT) and dF/dF HLOs after vehicle, Sota or Empa treatments. (B) Western blot results of SGLT1 levels in dF/dF HLOs after vehicle (Fsk only) or PHL treatments.

Table S1. List of selected SGLT inhibitors (adapted from Ref (12)).

Drug	Status	Selectivity SGLT2: SGLT1
Cana	FDA & EMA approved	~250-fold
Dapa	FDA & EMA approved	~1,200-fold
Empa	FDA & EMA approved	~2,500-fold
Sota	EMA approved	~20-fold
PHL	Not approved for clinical use	~ 1-fold
